# *Rhizobium* *moroccans* sp. nov., a Plant-Associated Bacterium from the Desert Medicinal Plant *Peganum harmala*, Reveals Genomic Adaptation to Arid Environments

**DOI:** 10.3390/microorganisms14040866

**Published:** 2026-04-11

**Authors:** Salma Mouhib, Khadija Ait Si Mhand, Juan Carlos Fernández-Cadena, Mohamed Hijri

**Affiliations:** 1 African Genome Center, University Mohammed VI Polytechnic (UM6P), Lot 660, Hay Moulay Rachid, Ben Guerir 43150, Morocco; salma.mouhib@um6p.ma (S.M.); khadija.aitsimhand@um6p.ma (K.A.S.M.); 2 OMICS Sciences Laboratory, Faculty of Health Science, Universidad Espíritu Santo, Samborondón 092301, Ecuador; jfernandezcadena@bwh.harvard.edu; 3 Brigham and Women’s Hospital, Harvard Medical School, Boston, MA 02115, USA; 4 Institut de Recherche en Biologie Végétale (IRBV), Département de Sciences Biologiques, Université de Montréal, 4101 Rue Sherbrooke Est, Montréal, QC H1X 2B2, Canada

**Keywords:** *Rhizobium moroccans*, bacterial endophytes, *Peganum harmala*, plant growth–promoting traits, whole genome sequencing, phenotyping

## Abstract

Members of the genus *Rhizobium* are best known for nitrogen-fixing symbioses with legumes, yet their diversity and evolutionary roles in non-legume hosts remain poorly explored, particularly in arid ecosystems. We report the isolation and characterization of strain AGC32, an endophytic bacterium obtained from surface-sterilized roots of the desert medicinal plant *Peganum harmala* collected in Moroccan drylands. Phylogenomic analyses placed AGC32 within the genus *Rhizobium* but clearly distinct from described species, with average nucleotide identity values below 96% and digital DNA–DNA hybridization values below 70%, supporting its designation as a novel species for which the name *Rhizobium moroccans* sp. nov. is proposed. Comparative genomics revealed extensive structural genome rearrangements relative to its closest sequenced relative, *Rhizobium deserti*, indicating a divergent evolutionary trajectory. The high-quality draft genome encodes metabolic pathways associated with adaptation to nutrient limitation and environmental stress, including complete allantoin utilization, polyphosphate metabolism, organic acid assimilation, and multiple systems involved in oxidative and osmotic stress tolerance. Phenotypic assays corroborated these genomic predictions, demonstrating the ability to metabolize diverse organic acids and carbohydrates and to express multiple plant growth–promoting traits, including nitrogen fixation and the solubilization of phosphorus, potassium, and silicon. Collectively, these findings expand the ecological and evolutionary diversity of *Rhizobium*, demonstrate its capacity to associate with non-legume medicinal plants in extreme environments, and highlight desert ecosystems as reservoirs of previously unrecognized microbial diversity with potential applications in sustainable agriculture in arid regions.

## 1. Introduction

Plant roots harbor highly structured microbial communities that play critical roles in nutrient acquisition, stress tolerance, and plant fitness. Within these communities, the root endosphere represents a strongly filtered niche enriched in bacterial taxa capable of intimate host colonization [1,2]. In arid and semi-arid ecosystems, this filtering is intensified by environmental stressors such as drought, salinity, and nutrient limitation, which shape both microbial diversity and functional traits [3,4]. Therefore, desert-adapted plants constitute promising reservoirs of stress-tolerant and metabolically versatile microorganisms [5].

Among root-associated bacteria, the genus *Rhizobium* is best known for nitrogen-fixing symbioses with legumes. However, accumulating genomic and ecological evidence indicates that *Rhizobium* species extend far beyond classical nodulating lifestyles [2]. Their multipartite genomes, comprising a conserved chromosome and accessory plasmids or chromids, facilitate horizontal gene transfer and ecological diversification [6,7]. Evolutionary analyses suggest that ancestral Rhizobiales were generalist root colonizers that later acquired nodulation genes, supporting repeated transitions between free-living, endophytic, and symbiotic states [8].

Consistent with this evolutionary plasticity, *Rhizobium* lineages are frequently detected in non-legume roots, including plants adapted to extreme environments. In Moroccan drylands, the root endosphere of *Malva sylvestris* L. was reported to be dominated by *Rhizobium* despite the absence of nodulation [2]. Similar enrichment of Rhizobiales has been documented in desert plants, where endophytic bacteria contribute to plant growth promotion and stress tolerance [3,9]. In non-legume hosts, rhizobia typically express plant growth–promoting traits, including phytohormone production, phosphate solubilization, siderophore secretion, and ACC deaminase activity, rather than symbiotic nodulation pathways [10,11,12]. These observations indicate that rhizobia occupy broader ecological niches than traditionally recognized.

Despite growing recognition of rhizobial ecological flexibility, the diversity and genomic basis of *Rhizobium* adaptation to non-legume medicinal plants in arid environments remain poorly characterized. Medicinal xerophytes are particularly relevant because their production of bioactive secondary metabolites may impose additional selective pressures on associated microbiota. *Peganum harmala* L., a perennial plant native to North African and Middle Eastern drylands, thrives in alkaline and drought-prone soils and synthesizes β-carboline alkaloids with documented antimicrobial activity [13]. While its phytochemistry is well studied, its endophytic bacterial diversity, especially rhizobial lineages, has not been taxonomically or genomically resolved.

We hypothesized that the root endosphere of *P. harmala* in Moroccan drylands harbors phylogenetically distinct and genomically adapted *Rhizobium* lineages reflecting both host filtering and extreme environmental pressures. Specifically, we expected that such isolates would (i) represent novel taxonomic entities within the genus and (ii) display genomic signatures associated with stress tolerance, metabolic versatility, and plant-associated lifestyles independent of nodulation.

To test this hypothesis, we isolated an endophytic *Rhizobium* strain from *P. harmala* roots and applied a polyphasic taxonomic framework integrating whole-genome sequencing, phylogenomic reconstruction, average nucleotide identity (ANI), digital DNA–DNA hybridization (dDDH), comparative genomic analysis, functional annotation, and phenotypic characterization. Through this integrative approach, we aimed to determine its taxonomic position and elucidate genomic features underlying adaptation to a non-legume host in an arid ecosystem.

Our findings support the designation of a novel species, *Rhizobium moroccans* sp. nov. AGC32, and expand the ecological and evolutionary understanding of rhizobia beyond classical legume symbiosis. This work highlights medicinal desert plants as reservoirs of previously unrecognized rhizobial diversity and provides insight into bacterial adaptation at the soil–root interface in extreme environments.

## 2. Materials and Methods

### 2.1. Plant Habitat and Isolation of Endophytic Bacteria

**Figure 1:**
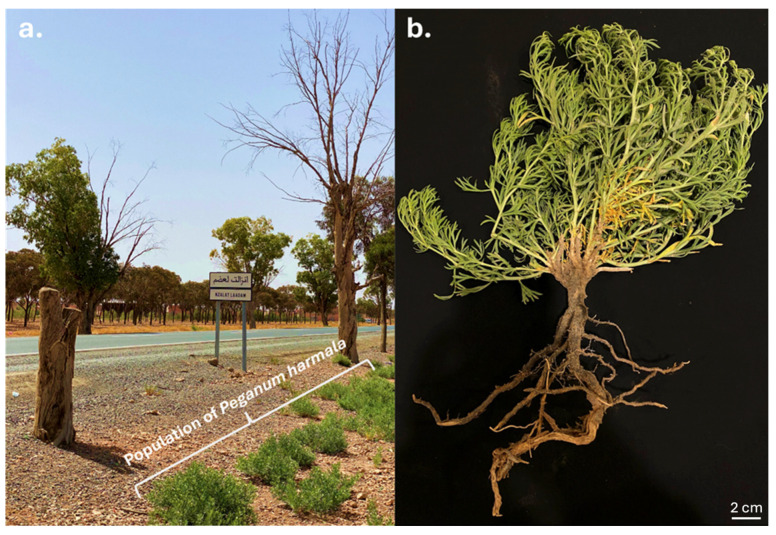
Sampling site of the host plant located in an arid environment along the road between Benguerir and Marrakech. (**a**) Natural population of *Peganum harmala* in its native arid habitat at the Nzalat Laadam site (Benguerir Province, Morocco). (**b**) Representative specimen collected from the site, showing both the root system and aerial parts of *P. harmala*. Scale bar: 2 cm.

Healthy individuals of *Peganum harmala* were collected on 27 May 2022 from a natural population in Nzalat Laadam, Benguerir, Morocco (32°06′49.6″ N, 7°57′13.0″ W) (Figure 1a). The site is characterized by arid climatic conditions with alkaline soils. Root samples were transported to the laboratory in sterile containers and processed within 24 h (Figure 1b). Surface sterilization, validation of sterilization efficacy, tissue maceration, serial dilution, plating conditions, colony purification, and long-term preservation procedures were performed as previously described by Mouhib et al. [14] (Figure S1). Briefly, sterilized root tissues were macerated under aseptic conditions and plated on tryptic soy agar (TSA) and potato dextrose agar (PDA). Plates were incubated aerobically at 28 °C for 48–72 h, and distinct colonies were purified by repeated streaking (Figure S1). The isolate designated AGC32 was preserved in 25% (vol/vol) glycerol at −80 °C.

### 2.2. DNA Extraction, 16S rRNA Gene Sequencing, and Whole-Genome Sequencing

Genomic DNA was extracted from overnight cultures grown in TSA broth at 28 °C using a protocol of Llop et al. [15]. DNA quality and concentration were assessed using a NanoDrop spectrophotometer (Thermo Fisher Scientific, Waltham, MA, USA) and agarose gel electrophoresis.

The 16S rRNA gene was amplified using universal primers pAF (5′-AGA GTT TGA TCC TGG CTC AG-3′) and 926R (5′-CCG YCA ATT YMT TTR AGT TT-3′) (Alpha DNA, Montreal, QC, Canada) under standard PCR conditions in a Mastercycler X50s (Eppendorf, Hamburg, Germany). The PCR program consisted of an initial denaturation at 95 °C/5 min, followed by 30 cycles of denaturation at 94 °C for 30 s, annealing at 58 °C for 30 s, and extension at 72 °C for 1 min. Each reaction had a total volume of 50 μL and was prepared using the *Taq* DNA Polymerase kit (Qiagen, Global Diagnostic Distribution, Témara, Morocco) according to the manufacturer’s instructions. The reaction mixture contained 1× reaction buffer, 2 mM MgCl_2_, 0.25 μM of each primer, 1 U of *Taq* polymerase, and 1.5 μL genomic DNA template from each isolate (approximately 10–20 ng of DNA). PCR products were purified and sequenced by a commercial service at the Centre d’Expertise et de Service (Genome Quebec, Montreal, QC, Canada). Sequence identity was determined using BLASTn (https://blast.ncbi.nlm.nih.gov, accessed on 2 April 2026) against the NCBI database [14] via Geneious Prime v2023.0.3 (Biomatters, Auckland, New Zealand).

Based on 16S rRNA gene affiliation and colony morphology, isolate AGC32 was selected for whole-genome sequencing using Illumina NextSeq 550 instrument (Illumina, Inc., San Diego, CA, USA), with a cluster density of 202 K/mm^2^ [14] (Figure S2).

### 2.3. Genome Assembly and Taxonomic Analysis

Raw reads were quality-checked using FastQC v0.11.9 and trimmed using Trimmomatic v0.39 with default parameters [16]. The overall bioinformatics workflow, including preprocessing, quality control, assembly, and taxonomic assignment, is summarized in Figure S2b and follows the pipeline previously described by Mouhib et al. [14] for foliar endophytes of the same host plant. The specific tools, parameters, and thresholds applied in the present study are detailed below.

Genome-based taxonomic analysis was performed using the Type (Strain) Genome Server (TYGS) [17] and PubMLST at https://pubmlst.org [18]. Digital DNA–DNA hybridization (dDDH) values were calculated using the Genome-to-Genome Distance Calculator implemented in TYGS, and average nucleotide identity (ANI) was computed using FastANI v1.33. Species delineation thresholds were applied as described by Mouhib et al. [14].

Phylogenomic relationships were inferred using 13 complete genomes of closely related *Rhizobium* type strains, including strain AGC32. An alignment-free phylogenomic analysis was conducted using SANS v2.5 [19]. Whole-genome assemblies were compared at the nucleotide level by computing shared and unique k-mers (k = 31). The phylogenetic signal was inferred from weighted k-mer–derived splits without sequence alignment. Only splits compatible with a strict tree topology were retained. Node robustness was assessed by bootstrap resampling of k-mers (1000 replicates), retaining splits with bootstrap support ≥ 0.75. The resulting strict consensus tree was exported in Newick format. *Mesorhizobium jarvisii* served as the outgroup. The tree was visualized and manually rooted using Geneious Prime v2023.0.3 (Biomatters Ltd., Auckland, New Zealand) and annotated with the GenBank accession numbers of each strain retrieved from NCBI.

### 2.4. Functional Annotation, Genome Visualization, and Comparative Genomics

Genome annotation was performed using Prokka [20]. Functional assignments were obtained using KEGG Orthologs via KofamKOALA [21] and a subsystems platform [22]. Metabolic pathways and gene counts were grouped into major functional categories and visualized as heatmaps.

Genes associated with endophytic adaptation and plant interaction were curated based on published datasets [23,24], including genes involved in amino acid metabolism, aromatic compound degradation, carbohydrate utilization, energy production, inorganic ion metabolism, metal resistance, lipid cofactors, nucleic acid metabolism, and stress response. Gene counts were normalized using Z-scores and visualized using RAWGraphs 2.0 as a red–blue heatmap.

Secondary metabolite biosynthetic gene clusters (BGCs) were predicted using antiSMASH with default parameters for the detection of NRPS, PKS, and RiPP clusters. The draft genome was visualized as a circular map using Proksee [25], displaying coding sequences, GC content, GC skew, genomic islands, mobile genetic elements, and antimicrobial resistance genes. Cross-referencing of mobile elements and resistance determinants was performed using MobileOG-db [26] and the CARD database [27].

Pairwise whole-genome alignments were conducted using progressiveMauve with default scoring parameters and the “Move contigs” option enabled. High-weight locally collinear blocks were analyzed to assess synteny conservation and genome rearrangements. The draft assembly of AGC32 was aligned with its closest related species, and locally collinear blocks were visualized using the Mauve viewer and exported as PNG images.

### 2.5. Phenotypic Profiling

Phenotypic characterization was performed using the Biolog GEN III MicroPlate system (Biolog Inc., Hayward, CA, USA) according to the manufacturer’s instructions. The system assesses utilization of 71 carbon sources and resistance to 23 chemical stressors through tetrazolium-based colorimetric reactions. Plates were incubated at 28 °C for 72 h, and absorbance was measured at 590 nm using the Biolog MicroStation™ reader (Biolog Inc., Hayward, CA, USA). Results were interpreted using the GEN III species database.

### 2.6. Plant-Growth-Promoting (PGP) Characterization

The plant growth-promoting traits were evaluated using qualitative assays as part of an initial screening approach. Strain AGC32 was cultured on TSA for 24 h at 28 °C [28]. Phosphate solubilization was assessed using the National Botanical Research Institute’s phosphate (NBRIP) medium [29]. Potassium solubilization was evaluated on Aleksandrov medium containing mica as the potassium source [30]. Zinc and silicate solubilization were tested on nutrient agar supplemented with ZnO or silicate, respectively, using bromothymol blue or bromocresol purple as pH indicators [31,32].

Plates were incubated at 28 °C for 5–7 days. Mineral solubilization was indicated by halo formation and color change surrounding bacterial growth. Nitrogen assimilation capacity was evaluated by streaking isolates onto nitrogen-deficient combined carbon (CC) medium [28]. Visible growth after 7 days at 28 °C was considered positive. All assays were performed in triplicate following the procedures described by Mouhib et al. [14].

## 3. Results

### 3.1. Isolation of Endophytic Bacteria

Surface-sterilized root tissues of *Peganum harmala* yielded several bacterial isolates on TSA (×0.1 and ×1) and a few on PDA (×0.1 and ×1). Among these, strain AGC32 was recovered from root fragments plated on PDA ×0.1, although this medium is typically used for fungal isolation. Colonies appeared after 48–72 h of incubation at 28 °C. They were circular, smooth, convex, opaque, creamy-white, and approximately 1 mm in diameter after 72 h (Figure S3). Cells were Gram-negative, motile coccobacilli, as observed by light microscopy, and no spore formation was detected.

### 3.2. Genome-Based Taxonomic Characterization

#### 3.2.1. Phylogenomics and Genome Structure

**Figure 2:**
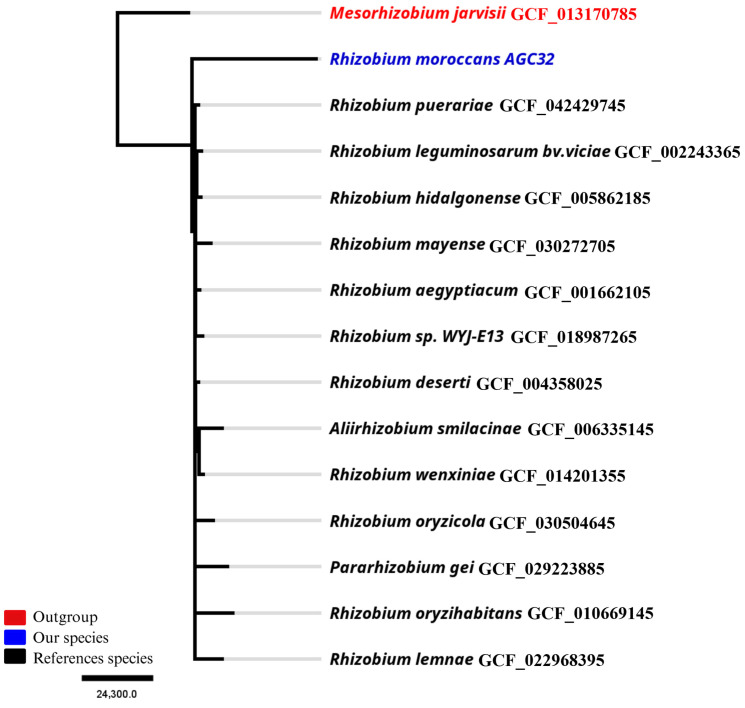
Rooted phylogenomic tree of the genus *Rhizobium* based on 13 complete genomes. Maximum-likelihood analysis positions isolate AGC32 (candidate *Rhizobium moroccans* sp. nov.) as a distinct lineage closely related to its nearest species. Colors indicate taxonomic grouping: red, outgroup (*Mesorhizobium jarvisii*); blue, the newly described species AGC32; black, reference species. The scale bar represents the number of substitutions per 100,000 nucleotide sites (scale = 24,300). Accession numbers of each strain are shown after the species name.

Phylogenomic analysis based on 13 complete *Rhizobium* genomes placed strain AGC32 within the *Rhizobium* clade and clearly separated from the outgroup *Mesorhizobium jarvisii* (Figure 2). AGC32 formed a distinct monophyletic lineage, separate from the closest type strains included in the analysis, including *Rhizobium deserti*, *Rhizobium puerariae*, *Rhizobium leguminosarum* bv. *viciae*, *Rhizobium hidalgonense*, *Rhizobium mayense*, and *Rhizobium aegyptiacum*.

Pairwise genome comparisons showed an average nucleotide identity (ANI) of 79.9% and a digital DNA–DNA hybridization (dDDH) value of 20% relative to its closest phylogenetic neighbor (Table S1).

**Figure 3:**
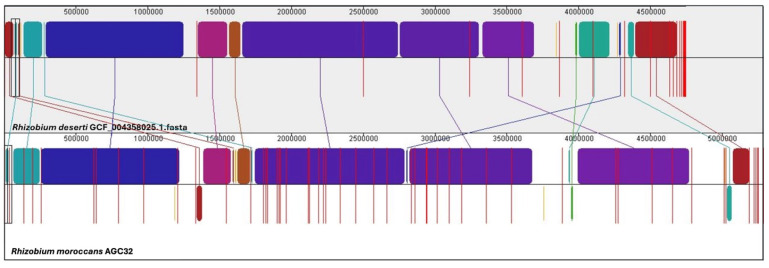
Comparative genomic synteny between *Rhizobium moroccans* AGC32 and *R. deserti*. Whole-genome alignment was performed using Mauve, highlighting significant genome rearrangements across 16 locally collinear blocks (LCBs; minimum weight = 13,100). Each colored block represents an LCB, corresponding to a homologous region conserved between the two genomes. Blocks of the same color indicate positional homology, while their vertical placement (above or below the central axis) reflects orientation relative to the reference genome (forward or inverted, respectively). Lines connecting blocks indicate conserved sequences between the genomes, illustrating inversions, translocations, and other structural variations.

Whole-genome alignment using progressiveMauve revealed partial conservation of the genomic backbone relative to *R. deserti*, with 16 locally collinear blocks (LCBs) (Figure 3). Several LCBs were inverted or repositioned. Increased structural variability was observed in terminal genomic regions.

#### 3.2.2. Genome Assembly and Quality Metrics

The draft genome of strain AGC32 consists of 5.24 Mb distributed across 55 contigs, with an N50 of 135,068 bp and a GC content of 61.62% (Table S2). Genome completeness was estimated at 98.82% with 0.92% contamination and an average sequencing coverage of 73×.

The partial 16S rRNA gene sequence showed 97.6% similarity to its closest validly named relative. Multilocus sequence typing (MLST) analysis did not match any existing allele profiles, and TYGS analysis did not affiliate AGC32 with any recognized type strain.

### 3.3. Functional and Metabolic Potential

#### 3.3.1. Functional Annotation

**Figure 4:**
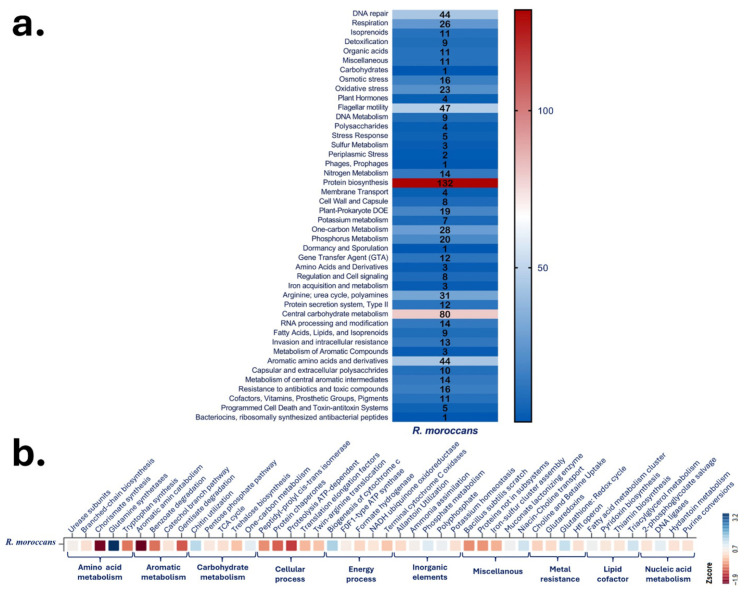
Functional and metabolic architecture of *R. moroccans* sp. nov. AGC32. (**a**) Distribution of functional capabilities based on KEGG ortholog (KO) gene counts grouped into major metabolic categories. (**b**) Z-score heatmap showing enrichment patterns across key metabolic subsystems, including amino acid metabolism, aromatic compound metabolism, carbohydrate metabolism, cellular processes, energy metabolism, inorganic ion metabolism, miscellaneous functions, metal resistance, lipid and cofactor metabolism, and nucleic acid metabolism.

Genome annotation identified genes distributed across major metabolic categories (Figure 4a and Table S3). The largest functional groups included protein biosynthesis (132 genes), carbohydrate metabolism (80 genes), flagellar motility (47 genes), and DNA repair (44 genes). Genes associated with oxidative stress, osmotic stress (16 genes), detoxification, and resistance to antibiotics and toxic compounds (16 genes) were also identified.

Subsystem annotation revealed genes involved in nitrogen metabolism (14 genes), iron acquisition, sulfur metabolism, and inorganic ion transport.

Heatmap analysis of normalized gene counts showed representation across amino acid metabolism, carbohydrate utilization, energy production, inorganic element metabolism, lipid metabolism, metal resistance, and nucleic acid metabolism (Figure 4b).

The genome encodes a complete denitrification pathway, high-affinity iron uptake systems (EfeUOB), glutamine synthetase variants, cytochrome c biogenesis proteins, glutathione redox components, and polyphosphate metabolism genes.

#### 3.3.2. Secondary Metabolite Biosynthetic Gene Clusters

**Figure 5:**
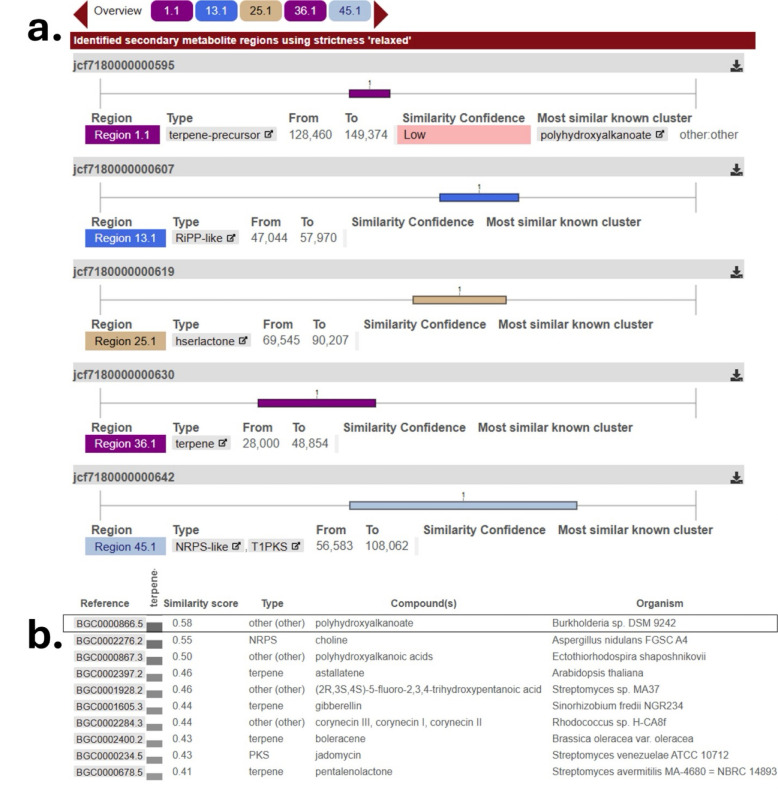
Genome mining and comparative analysis of biosynthetic gene clusters (BGCs) predicted using antiSMASH. (**a**) BGC regions identified in *R. moroccans* sp. nov. AGC32. (**b**) Top similarity scores of the predicted clusters compared to characterized BGCs in reference databases.

Genome mining using antiSMASH identified five putative secondary metabolite biosynthetic gene clusters (BGCs) distributed across the AGC32 genome (Figure 5). These included a terpene precursor cluster (Region 1.1), a RiPP-like cluster (Region 13.1), a homoserine lactone cluster (Region 25.1), a terpene cluster (Region 36.1), and an NRPS-like/Type I PKS hybrid cluster (Region 45.1). These BGC classes are commonly associated with membrane stabilization, oxidative stress protection, antimicrobial compound production, quorum sensing (AHL synthesis), and volatile organic compound biosynthesis in rhizobial species.

Similarity comparisons against the MIBiG database revealed generally low similarity scores (<0.6) to characterized clusters (Figure 5), with the highest similarity (0.58) observed for the polyhydroxyalkanoate-associated biosynthetic region. The limited similarity to known BGCs suggests potential novelty of secondary metabolite pathways in AGC32. Overall, the genome architecture of *Rhizobium moroccans* AGC32 is consistent with a free-living, rhizosphere-associated lifestyle typical of members of the genus *Rhizobium*.

### 3.4. Species Description

***Rhizobium moroccans*** **sp. nov.**

*Rhizobium moroccans* AGC32 was isolated from the roots of *Peganum harmala*.

Growth occurred on potato dextrose agar (PDA, ×0.1) at 28–35 °C within 72 h. Colonies were small (~1 mm in diameter), circular, raised, smooth, opaque, creamy-white, and mucoid, and had entire margins (Figure S3). Cells observed under light microscopy were Gram-negative and motile coccobacilli, and no spores were detected. All phenotypic assays were performed at ~28–30 °C under aerobic conditions.

**Figure 6:**
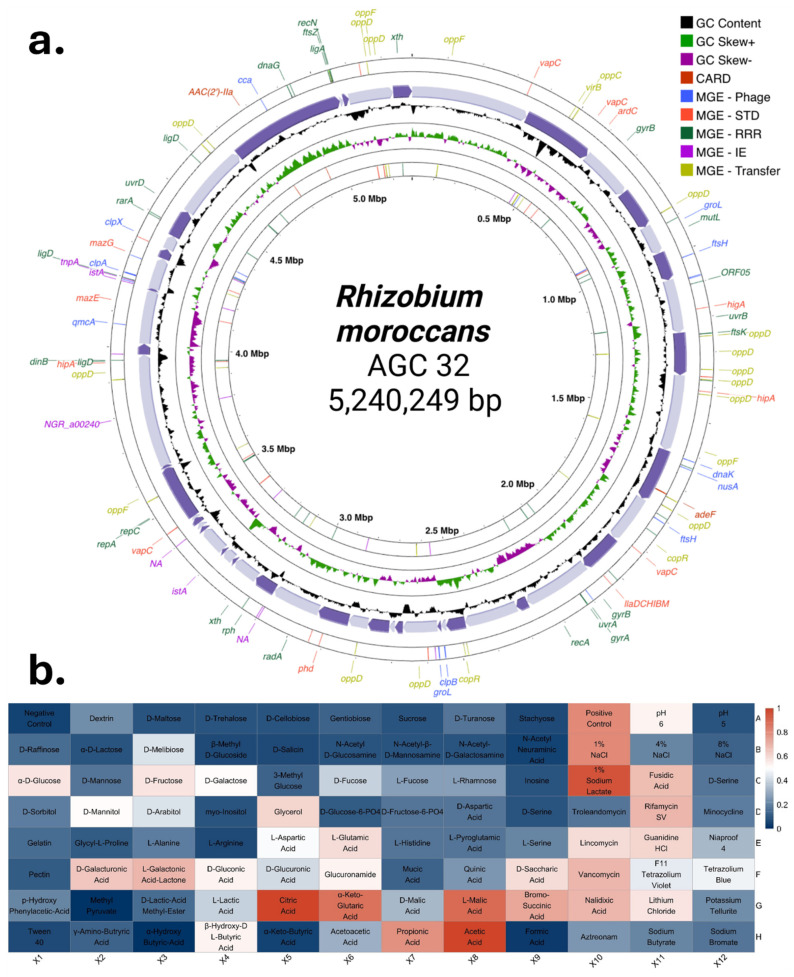
Circular genome map and phenotypic profiling of *R. moroccans* sp. nov. AGC32. (**a**) Circular genome visualization generated with Proksee, displaying GC skew, predicted genomic islands, and selected functional gene clusters. (**b**) Biolog GEN III metabolic profile showing carbon source utilization and chemical sensitivity patterns based on positive and negative reactions.

• Etymology: M.L. neut. adj. *moroccans*; “Of Morocco,” referring to the country from which it was isolated.
• Species name: *Rhizobium moroccans* sp. nov.
• Type strain: AGC 32^T^ (=CCMM B1344^T^).
• Accession numbers: 16S rRNA gene, PV739404; whole genome, SRR29855735.
• BioProject: PRJNA1133887; Biosample: SAMN43406629.
• DNA GC content: 61.62%.
• Genome size: 5.24 Mb (Figure 6a).
• ANI/closest relative: 79.9%.
• dDDH: 20%.

### 3.5. Phenotypic and Plant Growth-Promoting Traits

Following its designation as a novel species, strain AGC32 was phenotypically characterized using the Biolog GEN III MicroPlate system (Figure 6b). The metabolic profile generated was borderline, yielding mostly weak or ambiguous reactions, thereby preventing assignment to any species within the GEN III database. For routine cultivation, the strain was resuscitated on yeast extract mannitol (YEM) agar supplemented with 0.0025% Congo red. The isolate was aerobic and oxidative, exhibiting a chemoorganotrophic metabolism with broad utilization of carbohydrates and organic acids typical of rhizosphere-associated *Rhizobium* species.

In the chemical sensitivity panel, AGC32 grew at pH 5–6 and tolerated up to 1% NaCl. It metabolized multiple carbohydrates, including D-glucose, D-mannose, D-fructose, D-maltose, D-trehalose, D-cellobiose, sucrose, and dextrin. The organic acids utilized include citrate, D- and L-malate, propionate, acetate, formate, and α-ketoglutarate. The strain also assimilated several amino acids, including L-alanine, L-arginine, L-aspartate, L-glutamate, and γ-aminobutyric acid, as well as D-galacturonic acid, L-galactonic acid lactone, and glucuronamide.

Sensitivity to rifamycin SV, vancomycin, minocycline, and nalidixic acid was observed. Genome analysis revealed no virulence loci, toxin genes, or pathogenicity islands typically associated with opportunistic plant or human pathogens.

**Figure 7:**
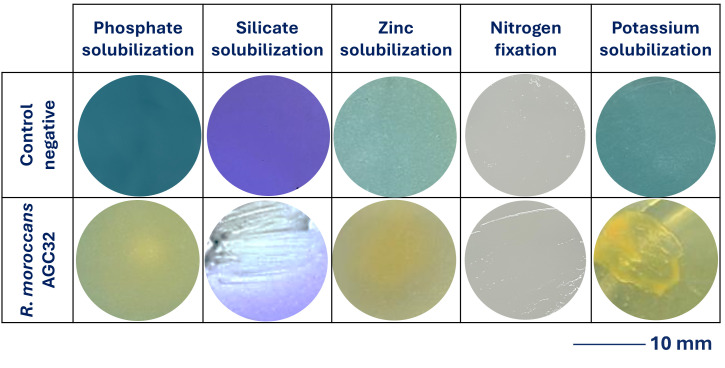
Plant growth-promoting traits of *R. moroccans* sp. nov. AGC32. Qualitative assays showing solubilization of phosphate, silicate, zinc, and potassium, as well as growth under nitrogen-limited conditions (columns). Rows represent the negative control and strain AGC32. Solubilization of inorganic elements was confirmed by color change in the medium and the formation of visible halo zones around colonies. Scale bar: 10 mm.

Qualitative plant growth-promoting (PGP) assays demonstrated growth under nitrogen-limited conditions and solubilization of potassium, silicate, phosphate, and zinc, indicated by halo formation and color change on respective media (Figure 7). These phenotypic traits are consistent with the genomic detection of genes involved in nitrogen metabolism, phosphorus metabolism, potassium homeostasis, and organic acid production (Figure 4a).

## 4. Discussion

Integrative genomic, functional, and phenotypic analyses support the classification of *Rhizobium moroccans* sp. nov. AGC32 as a novel endophytic bacterium associated with *Peganum harmala* roots. Phylogenomic reconstruction places AGC32 as an independent lineage within the *Rhizobium* clade, distinct from *R. deserti*, *R. puerariae*, and *R. leguminosarum*. Genome-based indices (ANI 79.9%, dDDH 20%, absence of MLST and TYGS matches) are consistent with species-level divergence according to current prokaryotic taxonomy standards [33]. These findings, together with its isolation from root tissues, are in line with previous reports indicating that members of the genus *Rhizobium* may occur as non-nodulating endophytes in non-legume hosts [34,35].

The genomic features of AGC32 provide insights into its possible ecological adaptation to arid environments, although these inferences remain based on sequence data. The genome encodes genes involved in core cellular processes such as protein biosynthesis, carbohydrate metabolism, DNA repair, and responses to oxidative and osmotic stress. Such features are commonly reported in soil and plant-associated bacteria inhabiting environments characterized by high irradiance, salinity, and water limitation [36]. In addition, the presence of genes related to motility and membrane transport systems may suggest the capacity for environmental sensing and interaction with the rhizosphere, although these traits were not directly assessed *in planta* [37].

The relatively limited representation of pathways associated with the degradation of complex aromatic compounds and certain amino acid derivatives may reflect a degree of metabolic specialization. This could be consistent with adaptation to nutrient-limited environments, where reliance on a restricted range of substrates, potentially including plant-derived compounds, may reduce metabolic costs [38]. Similarly, the presence of an allantoin utilization operon suggests a possible capacity to exploit nitrogen-containing compounds commonly found in root-associated environments, although its ecological relevance in situ remains to be determined [39,40].

Several genomic features are often associated with plant-associated lifestyles, including genes related to nitrogen fixation (*nifHDK*), phosphate transport (*pstSCAB*), oxidative stress response (*katG*, *katE*), and transport systems for organic compounds. While these elements indicate a genetic potential for nutrient acquisition and stress tolerance, their functional expression and contribution to plant performance cannot be inferred without experimental validation under relevant conditions. The relatively high proportion of genes assigned to transport, redox processes, and stress-related functions may nevertheless suggest a degree of ecological flexibility, as proposed by the “gene abundance–niche breadth” hypothesis [41].

Phenotypic profiling using Biolog assays indicates a strictly respiratory, chemoorganotrophic metabolism with the ability to utilize a range of carbohydrates, organic acids, and amino acids. The utilization of compounds such as citrate, malate, α-ketoglutarate, and γ-aminobutyric acid is consistent with substrates commonly reported in root exudates. However, these observations should be interpreted cautiously, as *in vitro* substrate utilization does not necessarily reflect substrate availability or ecological function in the rhizosphere.

The genome of AGC32 also contains several biosynthetic gene clusters (BGCs), including those putatively related to polyhydroxyalkanoates, terpenes, NRPS-like, RiPP-like, and polyketide compounds. While such clusters may indicate the capacity to produce secondary metabolites involved in microbial interactions or stress responses, their products and biological activities remain unknown. The relatively low similarity to characterized clusters (MIBiG < 0.6) suggests possible novelty, but experimental characterization is required to confirm their function.

The isolation of AGC32 from a non-legume host further supports the view that *Rhizobium* species may occur as facultative endophytes beyond classical symbiotic nodulation systems [42]. Although the combined genomic and phenotypic data suggest multiple traits that could be relevant for plant association, their actual roles in plant growth, stress tolerance, or ecological fitness remain to be demonstrated through controlled *in planta* experiments or field studies. At this stage, AGC32 can therefore be considered a candidate endophyte with genomic features compatible with a plant-associated lifestyle rather than a confirmed plant growth-promoting bacterium.

Overall, this study highlights the taxonomic novelty of *R. moroccans* sp. nov. and underscores the potential of medicinal plants as reservoirs of previously undescribed microbial diversity. Future investigations integrating transcriptomics, metabolomics, and targeted functional assays will be necessary to validate gene expression, characterize secondary metabolites, and clarify the ecological role and potential applications of this strain in agriculture or biotechnology [43].

## 5. Conclusions

This study provides evidence that the root endosphere of *Peganum harmala* in Moroccan drylands harbors a phylogenetically distinct and genomically adapted *Rhizobium* lineage potentially shaped by both host-associated factors and arid environmental conditions. In this context, strain AGC32 represents a novel taxonomic entity within the genus (*Rhizobium moroccans* sp. nov.), supported by phylogenomic analyses and genome-based metrics.

The genomic and phenotypic characterization of *R. moroccans* sp. nov. suggests the presence of traits commonly associated with stress tolerance, metabolic versatility, and a plant-associated lifestyle independent of nodulation. However, these features are inferred from genomic data and *in vitro* assays, and their functional expression and ecological relevance remain to be demonstrated under *in planta* or field conditions.

Comparative phylogenomics supports the species-level divergence of AGC32, while genome annotation reveals signatures that may be consistent with adaptation to arid soils and root-associated environments. The detection of putatively novel biosynthetic gene clusters further highlights the potential metabolic diversity of this strain, although their products and biological roles require experimental validation.

Overall, this work expands current knowledge of *Rhizobium* diversity in non-legume hosts and highlights medicinal plants as potential reservoirs of previously undescribed endophytic bacteria. Future studies integrating functional assays and multi-omics approaches will be essential to clarify the ecological role and potential applications of this strain.

## Data Availability

Accession numbers for the 16S rRNA gene sequences are available in GenBank under PV739404. Genome sequence data for the bacterial isolates have been deposited in the Sequence Read Archive (SRA) under SRR29855735. The type strain of the novel species has been deposited in the Moroccan Coordinated Collections of Microorganisms (CCMM) (https://www.ccmm.ma), with accession numbers B1344^T^ (*Rhizobium moroccans* sp. nov.). The genome assemblies are publicly accessible on Zenodo (https://zenodo.org) under the following DOIs: https://doi.org/10.5281/zenodo.18664957.

## Supplementary Materials

The following supporting information can be downloaded at: https://www.mdpi.com/article/10.3390/microorganisms14040866/s1, Table S1: Taxonomic identification of isolate AGC32 based on 16S rRNA gene sequencing using the Sanger method. Table S2: Summary of features of the draft genome assembly of endophytic isolate AGC32 from *Peganum harmala*. Table S3: Endophytic traits of strain AGC32 isolated from the root endosphere of *Peganum harmala*. Figure S1: Graphical representation of the workflow used for the isolation of bacterial endophytes from *P. harmala*. Figure S2: Experimental workflow and bioinformatic pipelines for whole-genome sequencing of endophytic bacteria isolated from *P. harmala* roots. Figure S3: Colony macro-morphology of isolate AGC32 grown on two different culture media.
