# Supplementary material for: Rhizobium moroccans sp. nov., a Plant-Associated Bacterium from the Desert Medicinal Plant Peganum harmala, Reveals Genomic Adaptation to Arid Environments
Source: Microorganisms. 2026 Apr 11;14(4):866. doi: 10.3390/microorganisms14040866 (PMC13119136; doi:10.3390/microorganisms14040866)
Supplement: Supplementary file 1 [file microorganisms-14-00866-s001.zip › microorganisms-4223999-supplementary.pdf]

SUPPLEMENTAL MATERIAL

Table S1. Taxonomic identification of isolate AGC32 based on 16S rRNA gene sequencing using the Sanger method.

Bacterial Species Names	Strain ID	Length (bp)	Identity (%)	Coverage (%)	Closest species (Accession number)	Accession number
Rhizobium moroccans	AGC32	762	97	100	*Rhizobium* sp. (PQ847472)	PV739404

Table S2. Summary of features of the draft genome assembly of endophytic isolate AGC32 from *Peganum harmala*.

**Bacterial Species**	*Rhizobium moroccans*
**Strain ID**	AGC32
**Assembler**	MaSuRCA
**Largest Contig**	526,861
**Number of contigs**	55
**N50**	135,068
**Genome Length**	5,240,249
**GC content (%)**	61.62
**Completeness (%)**	98.82
**Contamination (%)**	0.92
**Sequencing Depth (X)**	73
**Protein coding sequence (CDS)**	5,336
**tRNA aa**	20
**16S rRNA (%)**	93.3
**Accession number (SRA)**	SRR29855735

Table S3. Endophytic traits of strain AGC32 isolated from the root endosphere of *Peganum harmala*.

Traits	Proteins	*Rhizobium morocans*
Detoxification	2-dehydropantoate 2-reductase	☗
	glutathione S-transferase	☖
	S-(hydroxymethyl)glutathione dehydrogenase / alcohol dehydrogenase	☗
Plant polymer degradation/ modification	alpha,alpha-trehalase	☗
	cupin 2 domain-containing protein	☗
Redox potential maintenance	3-hydroxyisobutyrate dehydrogenase	☗
	acetoacetyl-CoA reductase	☗
	aldehyde dehydrogenase	☗
	malate dehydrogenase	☗
Secretion and delivery system	MFS transporter, DHA1 family, multidrug/chloramphenicol efflux transport protein	☗
	type VI secretion system lysozyme-related protein	☗
	type VI secretion system protein	☗
Transcriptional regulator	AraC/DeoR/Lrp/AsnC/LysR families of transcriptional regulators, or	☗
Transporter	ABC transport system ATP-binding/permease protein	☗
	branched-chain amino acid transport system ATP-binding protein	☗
	gluconate 2-dehydrogenase alpha chain	☗
	gluconate 2-dehydrogenase gamma chain	☖
	L-lysine exporter family protein LysE/ArgO	☗
	major facilitator superfamily domain-containing protein 12	☗
	major facilitator superfamily domain-containing protein 2B	☗
	MFS transporter, MHS family, metabolite:H+ symporter	☗
	NAD(P) transhydrogenase	☗
Heavy metal bioremediation	Cobalt-zinc-cadmium resistance protein / Nickel and cobalt resistance protein / Arsenate reductase / Copper resistance protein	☗
Hydrolytic enzymes	Phospholipase D / α-Amylase / Coccaine esterase / Proteases	☗
Indole acetic acid biosynthesis	Indole-3-glycerol phosphate synthase / Tryptophan synthase	☗
Oxidative stress and abiotic stress tolerance	Glutathione S-transferase / Hydroxyacylglutathione hydrolase / Glutathione synthetase / Superoxide dismutase / Polyphenol oxidase / Catalases / Trehalose-6-phosphate synthase, phosphatase / Betaine aldehyde dehydrogenase / Proline/betaine transporter	☗
Phosphorus availability	Pyrroloquinoline quinone biosynthesis / Alkaline phosphatase D / Polyphosphate kinase/ Exopolyphosphatase	☗
Salicylic acid biosynthesis	Isochorismate synthase / Chorismate synthase / Acetyl-coenzyme A carboxylase carboxyl transferase	☗
Sulfur availability and transport	Alkanesulfonate monooxygenase /Methanesulfonate monooxygenase / NADPH-dependent flavin mononucleotide reductase ArsH	☗
Temperature adaptation	Cold shock protein / Small heat shock protein / Heat shock protein / Heat-inducible transcription repressor	☗
Other	2-isopropylmalate synthase	☗
	diaminopimelate decarboxylase	☗

☖ Absence ☗ Presence

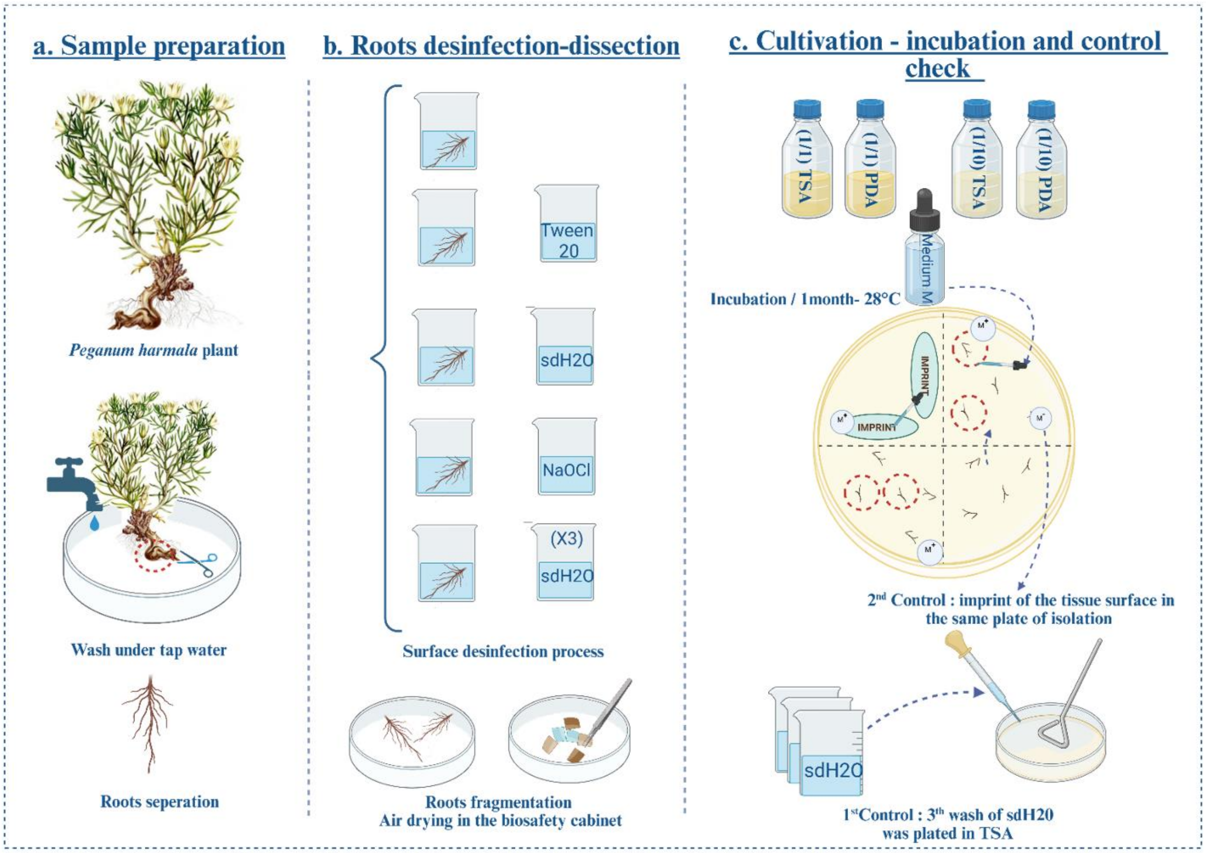

Figure S1. Graphical representation of the workflow used for the isolation of bacterial endophytes from *P. harmala*. (a) Sample preprocessing. (b) Surface sterilization and tissue fragmentation. (c) Cultivation, contamination assessment, and incubation. M+: TSA supplemented with M medium; M−: TSA without M-medium supplementation. Dashed circles on the Petri dishes indicate microbial growth surrounding the root fragments. An identical plate configuration was applied for the PDA medium.

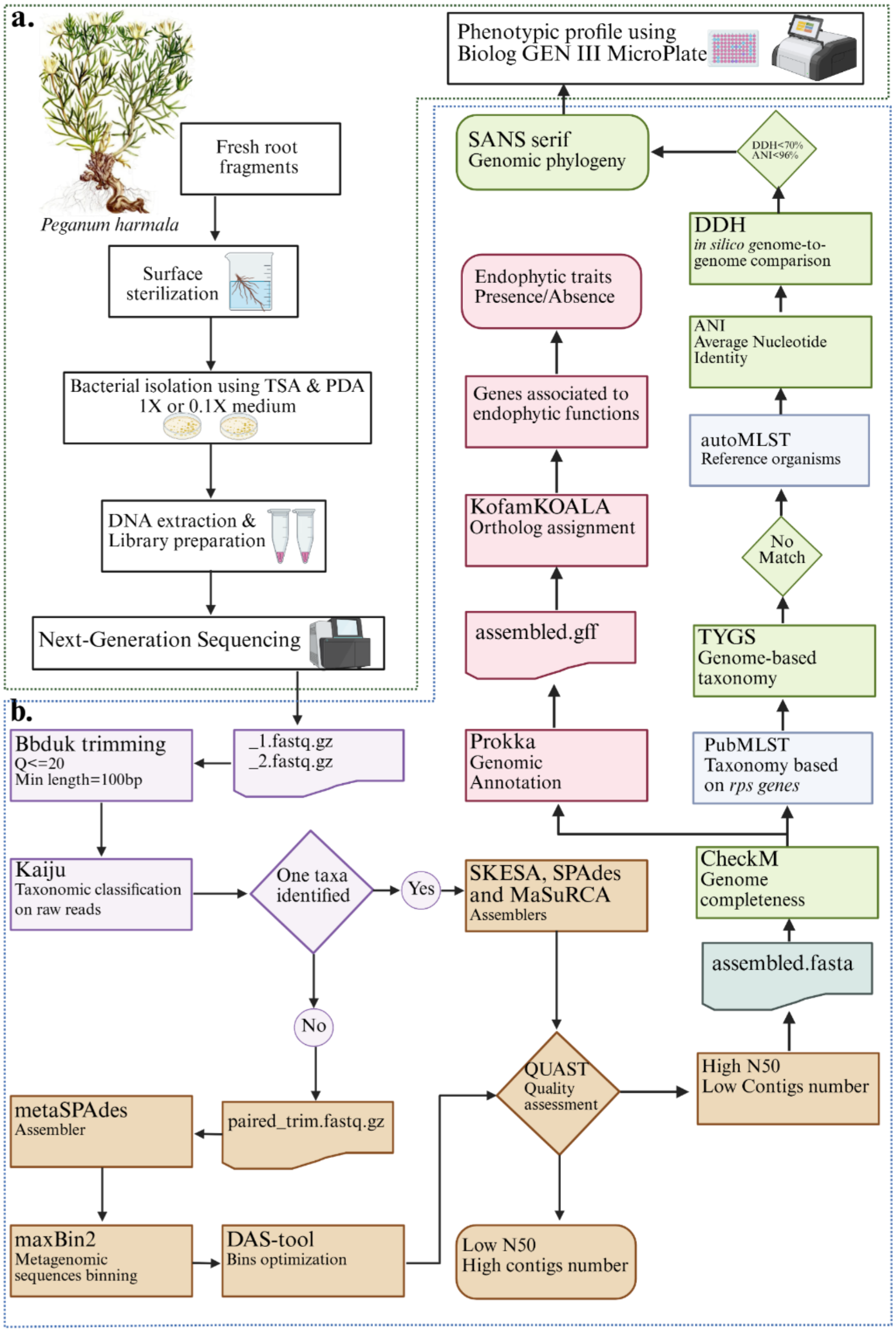

Figure S2. Experimental workflow and bioinformatic pipelines for whole-genome sequencing of endophytic bacteria isolated from *P. harmala* roots. (a) Bacterial isolation, genomic DNA extraction, and sequencing procedures. (b) Bioinformatic analysis and phenotypic profiling pipeline. Color codes indicate the different analytical stages: purple, pre-processing; brown, genome assembly and quality assessment; blue, molecular typing and characterization; green, genomic taxonomy and species delineation; red, functional annotation and trait mining.

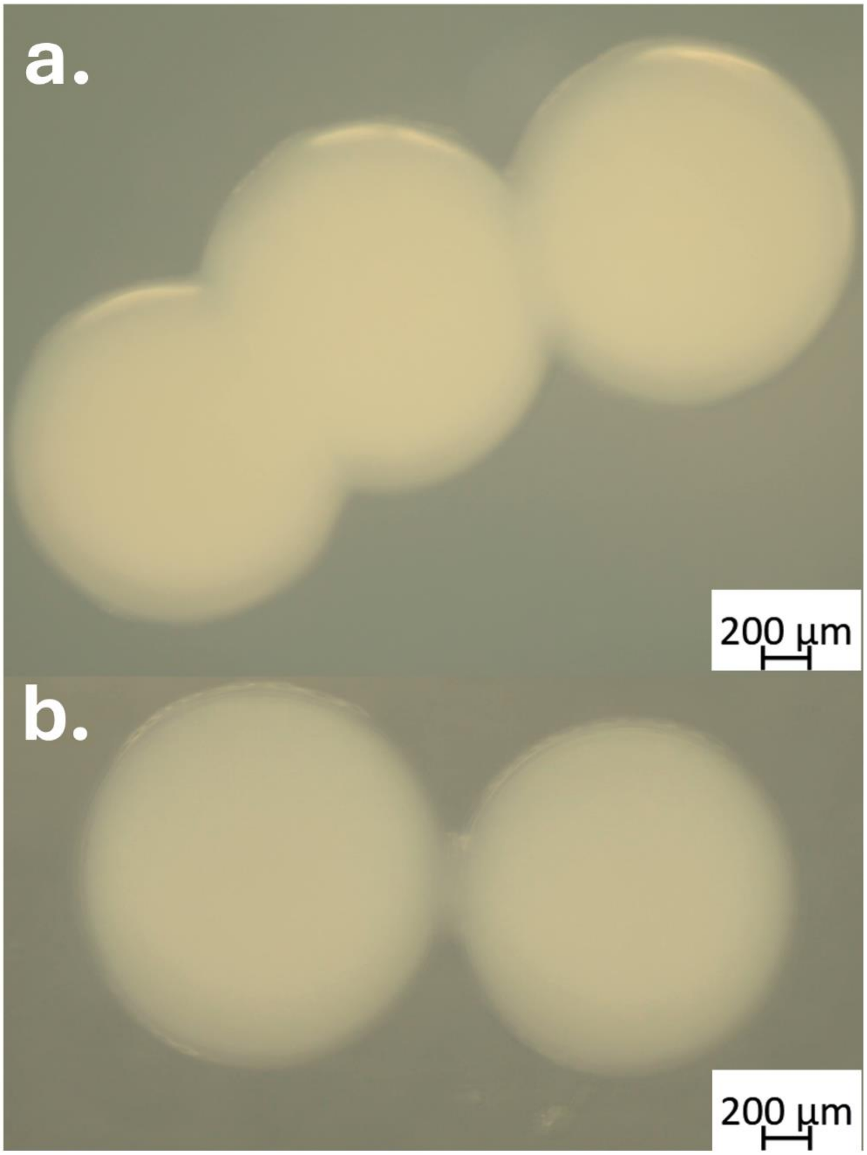

Figure S3. Colony macro-morphology of isolate AGC32 grown on two different culture media. Panels (a) and (b) display the colony morphology of *Rhizobium moroccans* sp. nov. strain AGC32 cultured on TSA (1×) and PDA (1×), respectively. Images were obtained using a stereomicroscope at 0.3× magnification.
